# Plenary 4 Reorienting health services: the transformational potential of health promotion

**DOI:** 10.1093/eurpub/ckac128.004

**Published:** 2022-10-25

**Authors:** Organised by: EuroHealthNet, Moderators: Martin Dietrich (EuroHealthNet), Caroline Costongs (EuroHealthNet)

## Abstract

Over the past decades, in Europe, the nature of our disease burden has shifted from more communicable and acute to chronic diseases such as cardiovascular diseases, diabetes, cancer and mental ill health. These diseases usually manifest themselves later in life, but they are not necessarily related to biological ageing. They mostly result from an accumulation of unhealthy living patterns since childhood and across the life course. Consumption of processed foods high in fat, salt, and sugar, smoking, excessive alcohol use, too little physical activity and too much stress all contribute to the growing and worsening burden of chronic diseases. These behaviours in turn are shaped by the social, environmental, cultural and economic conditions in which we live, grow, work and age.

Once they have developed, chronic diseases can be difficult, or even impossible to cure. Our health services, with a traditional curative approach, are not equipped for this chronic epidemic. There is an urgent need to shift our health services away from the predominant focus on cure and towards prevention, and for policy makers to invest in ensuring healthy living environments and societies. Health promotion and enabling people and population groups to increase control over their health, particularly those facing disadvantage, has the potential to transform our health services, and is critical to ensuring their resilience and sustainability.

Despite a growing awareness of the need for change, reorienting structures and systems in practice is challenging, as people can be resistant to change. Siloed approaches within the health sector, but also between social, health, and education sectors continue to prevail, and it is not always easy to find the right levers for change and to build bridges across administrations. This is compounded by a lack of infrastructure, organizational and workforce capacity for health promotion, and sustainable financing mechanisms. Much innovative work is however taking place, which we can learn from and scale up.

This plenary session will provide examples of different ways in which health-promoting approaches can reorient health services, strengthen health-promoting and community oriented primary care and prevent chronic diseases. It will highlight what we can learn from behavioural and cultural insights and social prescribing, as well as integrated community initiatives to further support people, across the social gradient, to lead and to maintain healthy lives. It will discuss target setting for further advocacy among policy makers.

**Speakers:**

**Rüdiger Krech**

WHO, Geneva, Switzerland

**Susan Michie**

University College London, UCL Centre for Behaviour Change, London, UK

**Jan De Maeseneer**

European Commission Expert Panel on Effective Ways of Investing in Health and Ghent University, Belgium

**Jet Bussemaker**

Council of Public Health & Society, Netherlands, Leiden University Medical Center, Leiden, Netherlands

**Cristiano Figueiredo**

USF da Baixa, Central Lisbon Health Centre Cluster, National School of Public Health, NOVA University Lisbon, Portugal

